# Dimensionless numbers to study cell wall deformation of stiff mutants of *Phycomyces blakesleeanus*

**DOI:** 10.1002/pld3.195

**Published:** 2019-12-27

**Authors:** Cindy M. Munoz, Joseph K. E. Ortega

**Affiliations:** ^1^ Department of Mechanical Engineering University of Colorado Denver Denver Colorado

**Keywords:** cell wall deformation, dimensionless numbers, *Phycomyces blakesleeanus*, stress relaxation, wall loosening, wall stiffness

## Abstract

The sporangiophores of *Phycomyces blakesleeanus* are large cylindrical aerial cells that elongate vertically at rates between 10 μm/min and 60 μm/min. Wild‐type sporangiophores grow toward light, opposed to gravitational acceleration and away from solid barriers (tropic responses). Sporangiophores of stiff mutants C149 and C216 exhibit diminished tropic (bending) responses. Originally, it was thought that the altered genes affect the “stiffness” (elastic wall deformation) of the cell wall. Subsequent investigations employing the pressure probe demonstrated that the irreversible (plastic) wall deformation was smaller for the stiff mutants compared to wild type and could account for the diminished tropic responses. However, it was not shown whether the elastic wall deformation was altered in these stiff mutants. Recent theoretical studies have identified dimensionless numbers that can be used to quantitate the magnitudes of biophysical processes involved in expansive growth of walled cells. In this study, dimensionless numbers are used to determine the magnitudes of elastic deformation rate, plastic deformation rate, and stress relaxation rate of the cell wall during expansive growth of the stiff mutant sporangiophores. It is found that the altered genes reduce stress relaxation rates and plastic deformation rates of the wall, but do not significantly alter the magnitude of the elastic deformation rates of the wall. These results indicate that the mutant genes reduce wall loosening chemistry in these sporangiophores and the genetic mutation is not expressed in a change in “wall stiffness,” but in “wall viscosity” or “wall extensibility.”

## 1 INTRODUCTION

Expansive growth and its regulation are central to the life and development of walled cells found in plants, algae, and fungi. Expansive growth and morphogenesis are regulated by controlling the cell wall mechanical properties which in turn are altered by the synthesis and incorporation of new wall materials and the chemical reactions inside the wall that produce wall loosening (Ortega, Truong, Munoz, & Ortega, 2015; Ortega, Truong, Munoz, & Ramirez, 2015). Learning how these cells regulate their mechanical properties to control growth and growth responses to environmental stresses and environmental stimuli can help with the development of novel uses for these cells in the biofuel, crop production, and pharmaceutical applications.

Walled cells of plants, algae, and fungi employ the same biophysical processes to produce expansive growth, that is water uptake and cell wall deformation. Water uptake occurs by osmosis and produces turgor pressure (*P*) which in turn stresses the cell wall. The wall stresses produce irreversible (plastic) and reversible (elastic) wall deformations (Ortega, Truong, Munoz, & Ortega, 2015; Ortega, Truong, Munoz, & Ramirez, 2015). Elastic wall deformation provides the resistance to produce and sustain large wall stresses and high turgor pressures (Ortega, Truong, Munoz, & Ortega, 2015; Ortega, Truong, Munoz, & Ramirez, 2015). The elastic properties are generally a function of cell wall structure and architecture. Plastic wall deformation produces a permanent increase in cell wall extension. It is generally regulated with biochemical reactions inside the wall that alter its mechanical properties by breaking load‐bearing bonds between wall polymers, that is wall loosening (Cosgrove, 2005, 2018). During plastic deformations, the cell wall maintains its integrity by simultaneously synthetizing and adding new cell wall material. In a turgid cell, wall loosening produces stress relaxation that reduces turgor pressure and initiates the uptake of water which in turn expands the cell wall to permanently increase cell volume (Cosgrove, 2005, 2018; Ortega, 2017).

Overall, the cell wall deformation behavior depends on the wall's composition, structure, architecture, and chemical reactions in the wall; thus, during expansive growth the mechanical properties are biochemically mediated (Ortega, Truong, Munoz, & Ortega, 2015; Ortega, Truong, Munoz, & Ramirez, 2015). Research has shown that the magnitude of expansive growth is sometimes regulated by the magnitude of turgor pressure (Green, 1968; Kroeger, Zerzour, & Geitmann, 2011; Lockhart, 1965; Ortega, Zehr, & Keanini, 1989; Proseus, Ortega, & Boyer, 1999; Proseus, Zhu, & Boyer, 2000). However, the mechanical properties of the wall control expansive growth during morphogenesis and tropic responses (Geitmann & Ortega, 2009; Ortega, 2012; Ortega, Munoz, Blakley, Truong, & Ortega, 2012; Ortega et al., 1991; Ortega, Truong, Munoz, & Ortega, 2015; Ortega, Truong, Munoz, & Ramirez, 2015; Probine, 1965; Taiz, 1984). Experimental evidence demonstrate that wall mechanical properties and behavior are modified by biochemical agents that loosen the wall (Cosgrove, 1993, 1999, 2000a, 2000b, 2001, 2018; Gerken, Donohoe, & Knoshaug, 2013; McQueen‐Mason, Durachko, & Cosgrove, 1992; Ortega, Truong, Munoz, & Ortega, 2015; Ortega, Truong, Munoz, & Ramirez, 2015; Palin & Geitmann, 2012; Park & Cosgrove, 2012; Proseus & Boyer, 2012a, 2012b). Biochemically mediated cell wall loosening is required for plastic deformation of the wall during expansive growth and the stress relaxation needed for water uptake and expansion of the cell volume (Cosgrove, 2015, 2018). For higher plant organs, such as stems, roots, and leaves, pH‐dependent proteins (expansins) have been shown to produce wall loosening and controlled polymer creep (time‐dependent deformation under an applied load) (Cosgrove, 1999, 2000a, 2000b, 2001; McQueen‐Mason et al., 1992). It is hypothesized that algal cells loosen their walls by breaking calcium bridges and reforming them between pectin polymers (Palin & Geitmann, 2012; Proseus & Boyer, 2012a; Proseus & Boyer, 2012a). It is unknown which biochemical agents regulate cell wall loosening in fungi, but some evidence exists that the mechanism might be similar to higher plants in that it is low pH‐mediated (Ortega, Truong, Munoz, & Ortega, 2015; Ortega, Truong, Munoz, & Ramirez, 2015).

Three interdependent biophysical equations that have been validated with experimental results and accurately model the expansive growth rate of walled cells have been termed by some as the Ortega Equations (Lewicka, 2006; Munoz, 2018; Pietruszka, 2013; Sridhar, Ortega, & Vernerey, 2018). Equation 1 describes the process of *net* water uptake in relative terms, where *v_w_* is the change in water volume in the cell, *L_w_* (*∆π*−*P*) is the volumetric rate of water uptake, and *v_T_* is the volumetric transpiration rate (Ortega, 2010; Ortega, Keanini, & Manica, 1988; Ortega & Welch, 2013). Cell wall deformation in relative terms is described by Equation 2, where *v_cw_* is the rate of change in volume of the cell wall chamber, *ϕ *(*P−P_c_*) is the irreversible (plastic) deformation rate, and (1/*ε*) d*P*/d*t* is the volumetric reversible (elastic) deformation rate of the cell wall (Ortega, 1985). Equation (1), (3) describes the rate of change of turgor pressure, d*P*/d*t* (Ortega, 2010; Ortega & Welch, 2013). These equations have been validated with in vivo creep and in vivo stress relaxation experiments conducted with the pressure probe (Cosgrove, 1985, 2005; Murphy & Ortega, 1995; Ortega, 2010; Ortega et al., 1988, 1991, 1989; Proseus et al., 1999, 2000)_._ Definitions for individual biophysical variables are found in Appendix S1.

(1) $$\nu_{w}=L_{w}\left(\Delta \pi -P\right)+\nu_{T}$$

(2) $$\nu_{\mathrm{cw}}=\phi \left(P-P_{c}\right)+\frac{1}{\varepsilon }\frac{dP}{dt}$$

(3) $$\left(\frac{1}{\varepsilon }\right)\frac{dP}{dt}=L_{w}\left(\Delta \pi -P\right)-\nu_{T}-\phi \left(P-P_{c}\right)$$

Dimensional analysis was conducted on the set of three biophysical equations, and seven dimensionless numbers were identified that represent the biophysical processes that control expansive growth rate (Ortega, 2016). These dimensionless numbers were used to provide a quantitative comparison of the magnitude of the biophysical processes that are involved in expansive growth rate of plant (*Pisum satinis* L.), fungal (*Phycomyces blakesleeanus*), and algal (*Chara corallina*) cells (Ortega, 2017, 2018, 2019). A recent review may be helpful for those not familiar with dimensionless Π parameters and their application to plant, algal, and fungal cells, (Ortega, 2018). Please refer to the Appendix S1 for biophysical variables definitions.

$$\prod_{\text{wv}}=\left(\frac{L_{w}P_{C}}{\nu_{s}}\right)=\left(\frac{\text{relative}\;\text{volumetric}\;\text{water}\;\text{uptake}\;\text{rate}}{\text{relative}\;\text{volumetric}\;\text{growth}\;\text{rate}}\right)$$

$$\prod_{\text{Tv}}=\left(\frac{\nu_{\mathrm{sT}}}{\nu_{s}}\right)=\left(\frac{\text{relative}\;\text{volumetric}\;\text{transpiration}\;\text{rate}}{\text{relative}\;\text{volumetric}\;\text{growth}\;\text{rate}}\right)$$

$$\prod_{\text{pv}}=\left(\frac{\phi P_{C}}{\nu_{s}}\right)=\left(\frac{\text{relative}\;\text{volumetric}\;\text{plastic}\;\text{deformation}\;\text{rate}\;\text{of}\;\text{the}\;\text{wall}}{\text{relative}\;\text{volumetric}\;\text{growth}\;\text{rate}}\right)$$

$$\prod_{\text{ev}}=\left(\frac{P_{C}}{\nu_{s}}\right)=\left(\frac{\text{relative}\;\text{volumetric}\;\text{elastic}\;\text{deformation}\;\text{rate}\;\text{of}\;\text{the}\;\text{wall}}{\text{relative}\;\text{volumetric}\;\text{growth}\;\text{rate}}\right)$$

$$\prod_{\text{we}}=\left(\frac{\varepsilon L_{w}}{\nu_{s}}\right)=\left(\frac{\text{relative}\;\text{volumetric}\;\text{water}\;\text{uptake}\;\text{rate}}{\text{relative}\;\text{volumetric}\;\text{elastic}\;\text{deformation}\;\text{rate}\;\text{of}\;\text{the}\;\text{wall}}\right)$$

$$\prod_{\text{Te}}=\left(\frac{\varepsilon \nu_{\mathrm{sT}}}{P_{C}\nu_{s}}\right)=\left(\frac{\text{relative}\;\text{volumetric}\;\text{transpiration}\;\text{rate}}{\text{relative}\;\text{volumetric}\;\text{elastic}\;\text{deformation}\;\text{rate}\;\text{of}\;\text{the}\;\text{wall}}\right)$$

$$\prod_{\text{pe}}=\left(\frac{\varepsilon \phi }{\nu_{s}}\right)=\left(\frac{\text{relative}\;\text{volumetric}\;\text{plastic}\;\text{deformation}\;\text{rate}\;\text{of}\;\text{the}\;\text{wall}}{\text{relative}\;\text{volumetric}\;\text{elastic}\;\text{deformation}\;\text{rate}\;\text{of}\;\text{the}\;\text{wall}}\right)$$

In this study, we are interested in learning what biophysical variables and processes change in stiff mutant sporangiophores that diminish their tropic (bending) responses. Recognizing that changes in water uptake and turgor pressure cannot cause bending of the sporangiophore (only asymmetric changes in biophysical properties of the cell wall can cause bending and changes in bending), this study focuses on the biophysical equation describing the biophysical extension and expansive growth of the cell wall (Equation 2) and the dimensionless numbers related to the cell wall, Π_pe_, Π_pv_, and Π_ev_. The cell wall dimensionless numbers (Π_pe_, Π_pv_, and Π_ev_) can be used to obtain the magnitudes of the plastic deformation rate, elastic deformation rate, and stress relaxation rate of the cell wall and compare their magnitudes to those of different cell species or mutants from same cell species. The magnitudes of the cell wall dimensionless numbers are determined by measuring the biophysical variables *ε*, *ϕ*, *P*_c_, and *v_s_* (the *steady* relative volumetric growth rate) with pressure probe experiments.

Here, Π_pe_, Π_pv_, and Π_ev_ parameters are determined and used to quantitate cell wall's stress relaxation rate, plastic deformation rate, and elastic deformation rate, respectively, of two stiff mutants (C216 and C149) of the fungal sporangiophores of *Phycomyces blakesleeanus*. The stiff mutants C216 and C149 are defective in the genes *madD*, *E*, *F*, *G*, and *J*. These mutants exhibit diminished tropic (bending) responses compared to the wild type (Campuzano, Galland, Alvarez, & Eslava, 1996; Grolig et al., 2000; Ootaki & Miyazaki, 1993). As a result, the mutants were termed “stiff” mutants, which indicate that the elastic deformation rate of the cell wall is reduced. Subsequent experimental research employing the pressure probe shows that the irreversible cell wall extensibility, *ϕ*, for stiff mutants C216 and C149 is significantly smaller than that of the wild type, but the magnitude of the critical turgor pressure, *P_c_*, is smaller as well (Ortega et al., 2012). The results indicate that the plastic deformation rate of the cell wall is reduced but the magnitude of the change could not be definitively determined. Here, in vivo turgor pressure step‐up experiments are conducted with the use of the pressure probe and used to determine *ε* (a measure of stiffness) of the stiff mutant sporangiophores so that the magnitudes of Π_pv_, Π_ev_, and Π_pe_ parameters can be determined. The values of *ε* are compared with those measured values from wild‐type sporangiophores in the same stage of development (Ortega, 2004, 2012). The average values of *ε* measured here, together with the published data from previous in vivo creep experiments (Ortega et al., 2012), are used to compute the magnitudes of Π_pv_, Π_ev_, and Π_pe_ for stiff mutants. The magnitude of Π_pe_ for wild‐type sporangiophores has been determined (Ortega, 2017, 2019). The average values of *ε*, *P*_c_, and *v*_s_ measured for wild type (Ortega, 2012; Ortega et al., 2012) are used to compute the magnitudes of Π_pv_ and Π_ev_ for wild type. The values of the dimensionless numbers obtained for the stiff mutants C216 and C149 are compared to the respective dimensionless numbers obtained from wild‐type sporangiophores. It is found that the magnitudes of *ε* of both stiff mutants C216 and C149 are statistically the same as those obtained for wild‐type sporangiophores and that the magnitudes of Π_ev_ for stiff mutants are slightly smaller than those of wild‐type sporangiophores. Thus, it is concluded that the mutant sporangiophores are not stiffer. In contrast, the magnitudes of Π_pv_ and Π_pe_ for C216 and C149 mutant sporangiophores are considerably smaller than those obtained for wild‐type sporangiophores. These results indicate that the wall loosening chemistry is significantly diminished in the stiff mutants. Furthermore, the results indicate that the term “stiff” mutant is not an appropriate indicator of the change of cell wall properties for mutant sporangiophores of C216 and C149 strains.

## 2 MATERIALS, METHODS, AND CALCULATIONS

### 2.1 Biological material

Vegetative spores of the stiff mutant gene strain C216 *geo− (−)* were originally obtained from Ishinomaki Senshu University, Miyagi, Japan. The C149 *madD120*(−) strain was obtained from ATCC: The Global Research Center, Virginia, USA. Sporangiophores were inoculated on sterile growth medium consisting of 4% (w/v) YM agar. After inoculating, the sporangiophores were incubated under diffuse incandescent light and constant temperature (22°C ± 2°). Stage IVb sporangiophores, 1.5–2.5 cm in length, were selected for experiments from the second to the seventh crop. In this study, stage IVb sporangiophores are used because they exhibit nearly constant growth rate and rotation (Bergman et al., 1969; Cerda‐Olmedo & Lipson, 1987).

### 2.2 Change in length

The change in length, *∆L*, in the sporangiophore is determined by measuring the change in length, *∆L,* of a reference point (the edge of the sporangium). The length is measured by taking snapshots using a USB camera (720P HD, Gucee HD92 Skype Web Camera) attached to a long focal length horizontal microscope (Gaertner; 7011Keyepiece and 32m/m EFL objective) mounted to a 3‐D micromanipulator (Line ToolCo.; modelH‐2, with digital micrometer heads). Snapshots were acquired using open‐source software Vividia Ablescope and were analyzed using open‐source software ImageJ. The diameter of the sporangium for each sporangiophore was measured before initiation of the experiment to use as a scale reference in ImageJ. Snapshots were acquired at 30‐s intervals for in vivo turgor pressure step‐up experiments.

### 2.3 Turgor pressure

The turgor pressure of the sporangiophore is measured continuously using a manual version of the pressure probe (Ortega et al., 1991, 1989). A USB gage pressure transducer is used in the pressure probe which was purchased from Ellison Sensors Inc (model GD4200 USB‐100BAR) and tested for calibration inside the pressure probe with a Heise Bourdon Tube Pressure Gauge (Dresser Industries; model CMM, 0–200 PSIG Range). The transducer's output was recorded on the software package included with the USB transducer.

**Figure 1:**
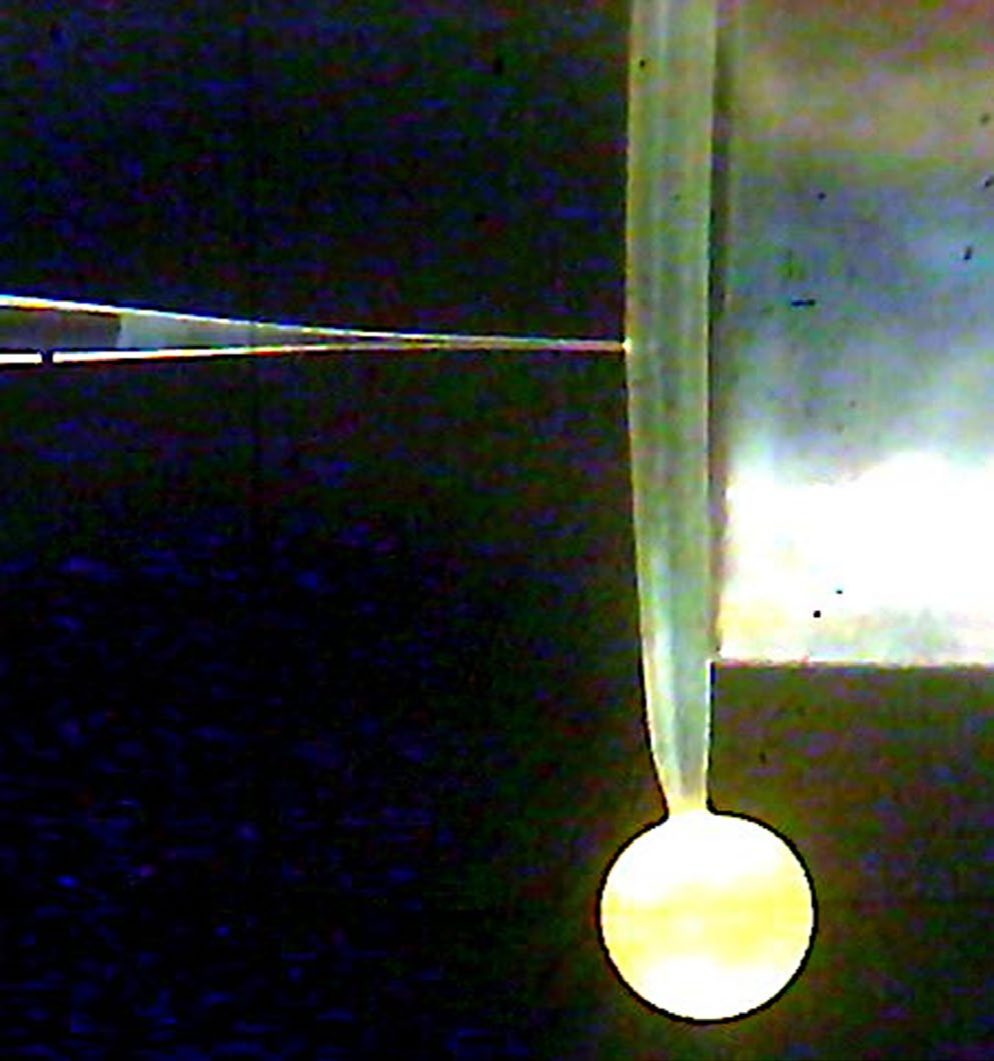
Snapshot of a sporangiophore being impaled by the microcapillary tip. The cell sap interface flows out of the cell (represented by the red arrow) and needs to be brought to the surface of the cell in order to read the turgor pressure and ensure oil flows into the cell vacuole. This is accomplished by increasing the pressure with the pressure probe

The pressure probe was mounted on a 3‐D manipulator so that the microcapillary tip (10–15 μm in diameter) could be guided to impale the sporangiophore under visual observation using a High‐Resolution Digital Microscope (Jiusion 40 to 1,000× Magnification Endoscope, 8 LED USB 2.0 Digital Microscope, Amazon). The microcapillary of the pressure probe was filled with inert silicon oil (Dow Corning Corp.; fluid 200, 1.5 centistoke viscosity). After the cell was impaled, the cell sap–oil interface was pushed to the surface of the cell vacuole and maintained at the fixed location to measure the turgor pressure of the sporangiophore (Ortega et al., 1991, 1989) (Figure 1). The higher turgor pressure was maintained by small injections of inert silicon oil and also ensuring silicon oil was flowing into the cell vacuole for immediate step‐up in turgor pressure.

### 2.4 In vivo turgor pressure step‐up experiment

The in vivo turgor pressure step‐up experiment requires constant short time interval (30 s) increases in turgor pressure of 0.03–0.04 MPa (turgor pressure step‐ups) produced to the sporangiophore and immediate measurement of the elongation. The biophysical variable, *ε*, can be determined by this method and through the Ortega Equation (Equation 2) rewritten for a cell that elongates in the longitudinal direction at finite time intervals, ∆*t*, Equation 2a (Ortega, 1985, 2012). When pressure step‐ups are large (*P* ≥ 0.02 MPa) and at short time intervals (30 s), it ensures that the irreversible component in Equation 2a is minimized and can be neglected.

(2a) $$\nu_{s}=\frac{1}{L}\frac{\Delta L}{\Delta t}=\phi \left(P-P_{C}\right)+\frac{1}{\varepsilon }\frac{\Delta P}{\Delta t}$$

To see this, Equation 2a is multiplied by ∆*t* and rearranged to solve for *ε*, Equation 2b.

(2b) $$\varepsilon =\frac{\Delta P}{\frac{\Delta L}{L}-\left\{\phi \left(P-P_{C}\right)\right\}\Delta t}$$

As the limit of ∆*t* approaches zero (for short time intervals), the term *ϕ *(*P*−*P*_c_) also approaches zero and becomes negligible. Equation 2b is then simplified to Equation 2c, where ∆*P* is the magnitude of the pressure step‐up, ∆*L* is the change in length after the pressure step‐up, and *L* is the initial length of the sporangiophore.

(2c) $$\varepsilon =L\frac{\Delta P}{\Delta L}$$

Furthermore, because the pressure step‐ups are larger than 0.02 MPa, the steady‐state growth rate (*ϕ *(*P*−*P*_c_) = steady‐state growth rate when *P* = constant) is reduced at the first pressure step‐up for the growing sporangiophores. This also makes the irreversible deformation term smaller and therefore negligible. This is validated by experimental research in which large turgor pressure step‐ups (*P* > 0.02 MPa) cause a transient decrease in elongation growth rate of sporangiophores of *P. blakesleeanus* (Ortega et al., 1991, 1989).

### 2.5 Protocol for in vivo turgor pressure step‐up experiments

**Figure 2:**
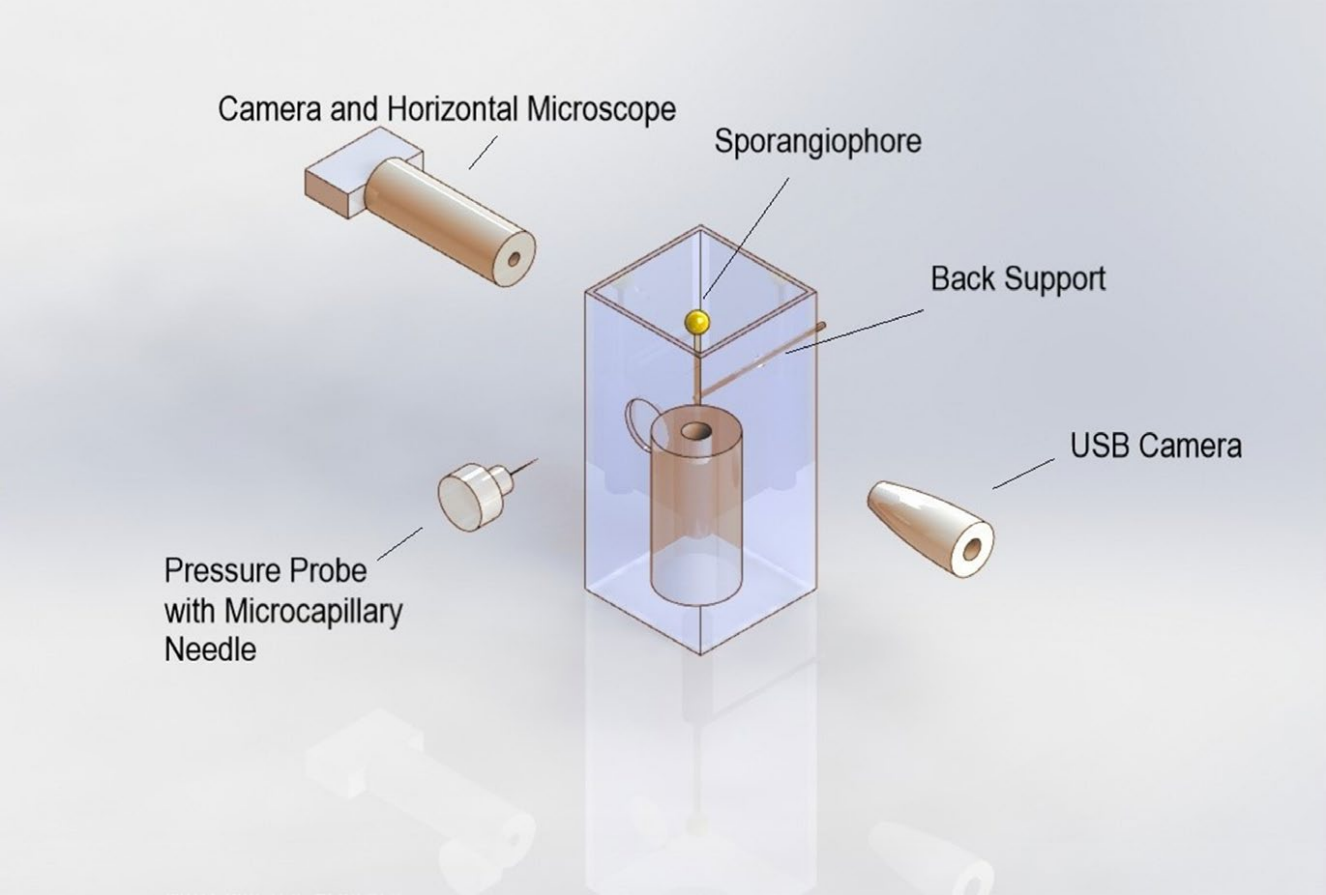
Experimental set‐up for an in vivo pressure step‐up experiment. A horizontal microscope with a USB camera is used to capture elongation of the sporangiophore. A USB microscope helps guide the microcapillary needle into the sporangiophore. A pressure probe is used to impale, measure, and control the cell's turgor pressure

**Figure 3:**
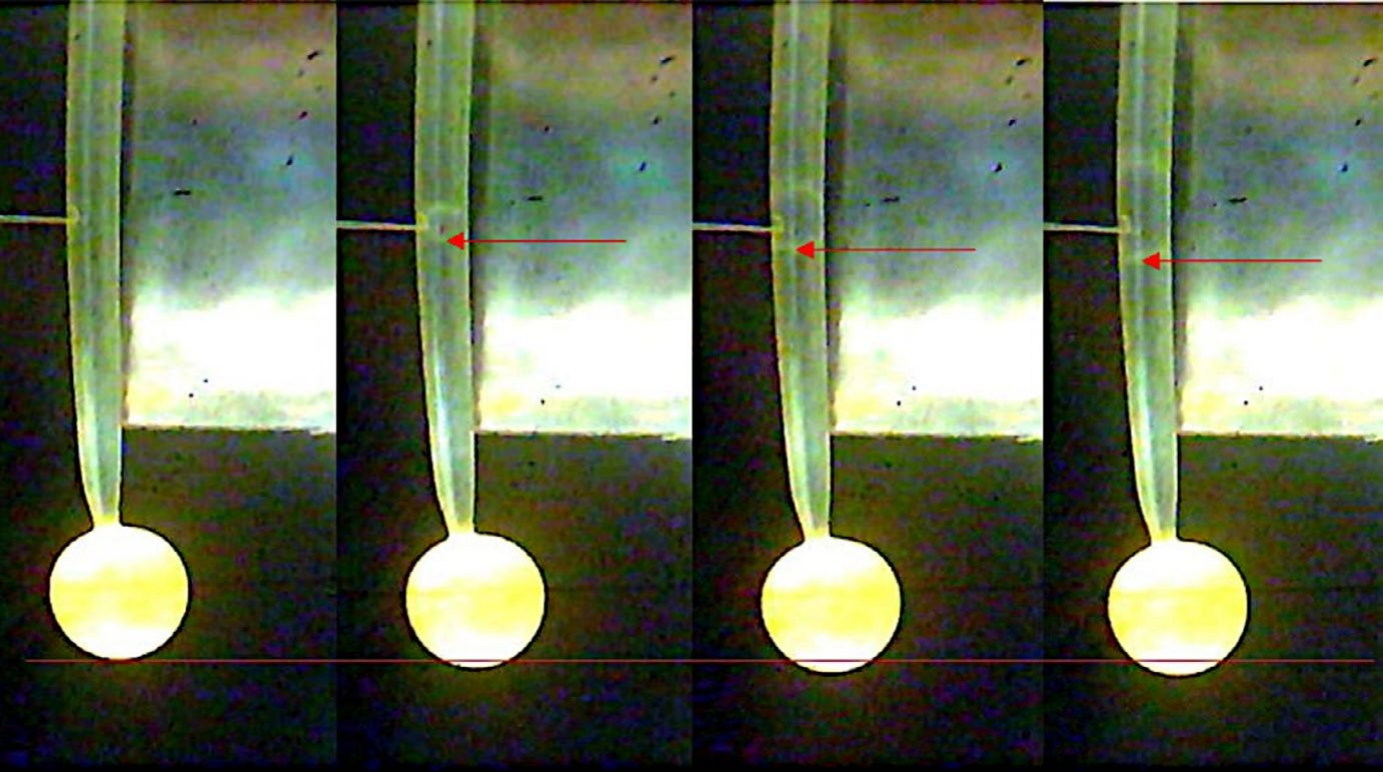
Snapshots of the sporangiophore as it is elongated by the addition of oil using a microcapillary attached to a pressure probe. The red arrow indicates oil being injected into the cell with each turgor pressure step‐up. The red line indicates the reference line used to measure the change in length of the cell after each turgor pressure step‐up. Images were analyzed using ImageJ

A stage IVb sporangiophore (typically 1.5–2.5 cm in length) in a glass shell vial is selected and adapted for 20 min to the room temperature of 21–23°C (Figure 2), to room lights (cool white fluorescent lamps hung from the ceiling), and to bilateral swan‐neck light guides (from Schoelly Fiberoptic; the end of each light guide is positioned approximately 8–12 cm on either side of the sporangiophore at an angle of about 30° from the horizontal) from a fiber‐optic illuminator (Flexilux 90; HLU Light Source 90/Wfrom Schoelly Fiberoptic, Denzlingen, FRG, which filtered out nearly all of the infrared light). The sporangium is measured and recorded using the micrometer attached to the USB camera microscope set‐up. Following this adaptation period, the length of the sporangiophore is measured and the pressure transducer software is initiated. The pressure in the pressure probe is increased between 0.03 and 0.09 MPa before impaling the cell. The cell is immediately impaled by the microcapillary tip of the pressure probe to measure the turgor pressure. Once a cell sap interface is located, the image acquisition software is also initiated to take snapshots of the sporangiophore at 30‐s intervals with a 5‐s delay from the pressure step‐ups. The snapshots are later analyzed to measure elongation using ImageJ (Figure 3). For the first 30–60 s into the experiment, the turgor pressure is maintained, 30 s after, the turgor pressure is increased by 0.03–0.04 MPa and maintained for 30 s by injecting inert silicon oil into the cell vacuole. This same procedure (turgor pressure step‐up) is followed until the cell ruptures by not sustaining any more oil into the vacuole and bursting or until a leak is present. Figure 2 shows the experimental set‐up for an in vivo turgor pressure step‐up experiment.

## 3 RESULTS

### 3.1 Longitudinal volumetric elastic modulus, ε

**Figure 4:**
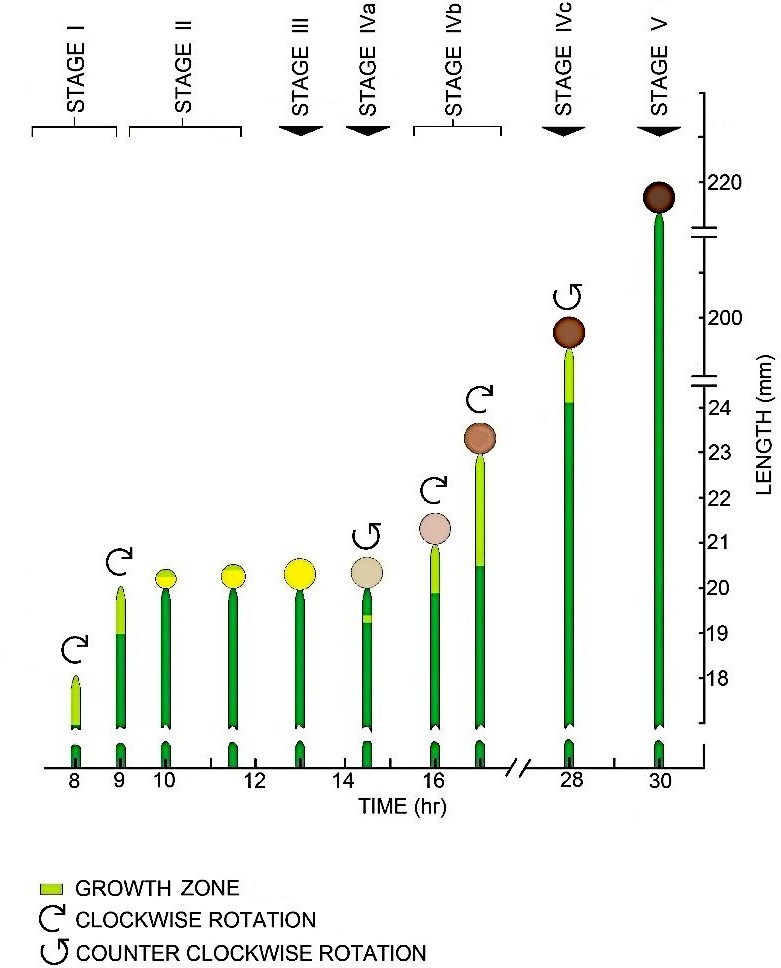
Developmental stages of the sporangiophore of *Phycomyces blakesleeanus*. The large cylindrical single‐celled sporangiophores are approximately 150 μm in diameter and can grow in length to ten centimeters or longer. Sporangiophore development is divided into five stages (stages I, II, III, IV, and V), and stage IV is further divided into three sub‐stages (IVa, IVb, and IVc). The sporangiophores exhibit expansive growth and irreversible wall deformation in a region termed the “growth zone.” The sporangiophores exhibit helical growth and reverse direction of helical growth depending on the stage. In stages I and II, the growth zone is located at the apical tip (tip growth) and adjacent to the sporangium (intercalary growth) in stage IV. Stage IVb (used in this study) exhibits nearly constant growth zone length, elongation growth rates, and rotation growth rates for many hours. The sporangiophores can detect many environmental stimuli such as gravitational acceleration, ethylene, mechanical stretch, gases, temperature, wind, light intensity, spatially asymmetric distribution of light, the presence of solid objects, and changes in turgor pressure (Bergman et al., 1969; Cerda‐Olmedo & Lipson, 1987; Ortega et al., 1989). Response to these stimuli is produced with symmetric and asymmetric changes in expansive growth rate (e.g., growth responses and tropic responses) (Bergman et al., 1969; Cerda‐Olmedo & Lipson, 1987; Ortega et al., 1989). Figure obtained from (Ortega et al., 2012)

The magnitude of the longitudinal volumetric elastic modulus, *ε,* was determined from in vivo turgor pressure step‐up experiments conducted on stage IVb (growing) sporangiophores (Figure 4) from stiff mutant strains, C216 *geo− (−)* and C149 *madD120 *(−). Stage IVb sporangiophores (growing sporangiophores) exhibit both irreversible and reversible cell wall deformation (Ortega et al., 2012) (Figure 4). Therefore, a specified protocol must be followed to ensure the elongations measured are purely elastic.

**Figure 5:**
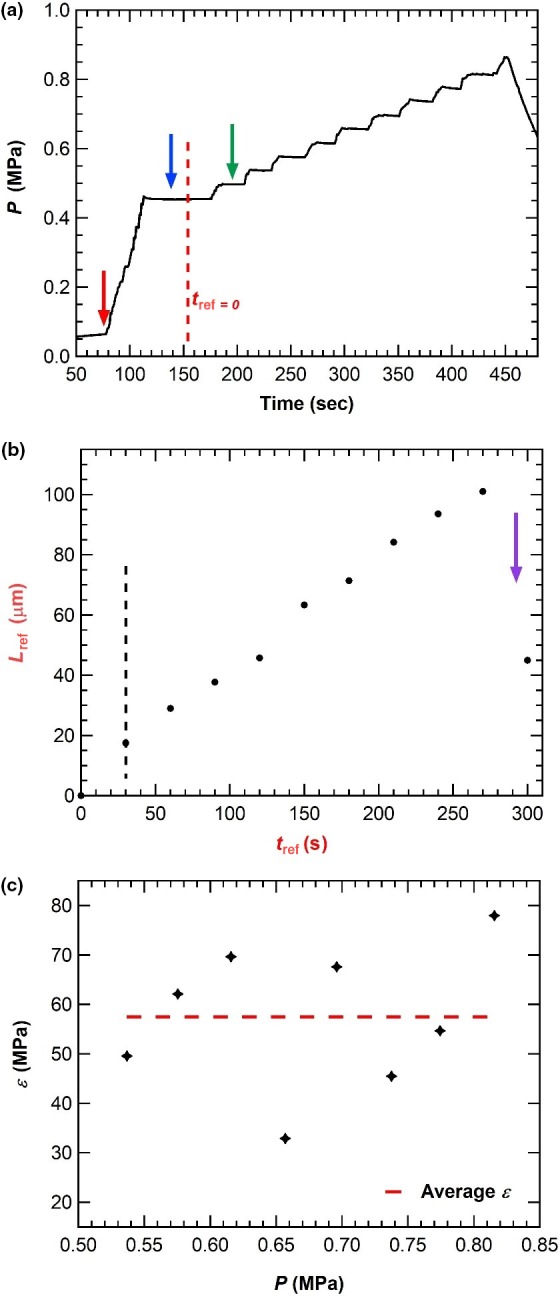
Turgor pressure, elongation, and longitudinal volumetric elastic modulus behavior for a single stage IVb C149 sporangiophore during an in vivo turgor pressure step‐up experiment. (a) Turgor pressure behavior. The cell is impaled at the first downward pointing red arrow, and the turgor pressure is increased until the cell sap interface is brought to the surface of the cell. Turgor pressure is maintained constant (blue arrow) by injecting inert silicon oil until the first pressure step‐up is applied to the cell (green arrow). The new turgor pressure is maintained constant until 30 s later and the next pressure step‐up is applied. *t*_ref=0_ indicates the time when length measurements due to pressure step‐ups began (L_ref_). *L*_ref_ excludes the initial length of the cell. A total of 8 pressure step‐ups were applied to the sporangiophore before a leak occurs. (b) Elongation as a function of time. Length (*L*_ref_) was graphed for the corresponding pressure step‐ups at 30‐s intervals. The cell sustains a maximum pressure of ≈0.83 MPa before a leak occurs. The leak is represented by a decrease in length (*L*_ref_) due to the leakage of oil and cell material (purple arrow). The change in length, ∆*L*_ref_, and change in turgor pressure, ∆*P*, are used to determine ε for each pressure step‐up. A total of 8 *ε*‐values are determined for this experiment, and an average value is computed. (c) *ε* as a function of turgor pressure, *P*. Each *ε*‐value is graphed with its corresponding pressure. The average ε‐value for this sporangiophore is represented as a red dashed line (*ε* = 57 MPa). The volumetric elastic modulus is approximately constant for the range of turgor pressures the cell was subjected to

A typical in vivo turgor pressure step‐up experiment for a stage IVb sporangiophore is shown in Figure 5 with 0.04 MPa pressure step‐ups. Turgor pressure step‐ups larger or equal than 0.03 MPa at short time intervals (30 s) ensures that the irreversible deformation rate (plastic deformation rate) on a growing sporangiophore is negligible and that all elongations produced after the first turgor pressure step‐up are purely elastic. Previous research on wild‐type sporangiophores of *P. blakesleeanus* demonstrated that turgor pressure step‐ups greater than 0.02 MPa (*P* > 0.02 MPa) produce a transient decrease in growth rate and larger steps‐up in turgor pressure produce larger decreases in growth rate and longer periods of decreased growth rate (Ortega et al., 1989). Taking this into account, the first pressure step‐up of a turgor pressure step‐up experiment employing a pressure step‐up greater or equal to 0.03 MPa results in an initial decrease in elongation growth rate for growing sporangiophores, making the plastic deformations after this first step‐up insignificantly small (see protocol for in vivo turgor pressure step‐up experiment for additional mathematical information). The plastic wall deformations are eliminated by subsequent turgor pressure steps‐up at short time intervals (30 s). This procedure is employed to ensure the elongations measured for a growing sporangiophore during a turgor pressure step‐up experiment are elastic deformations. Here, the first step‐up is neglected and only subsequent turgor pressure steps‐up are used to compute an *ε‐*value. A mean *ε‐*value for each sporangiophore tested is obtained by averaging all *ε‐*values.

**Figure 6:**
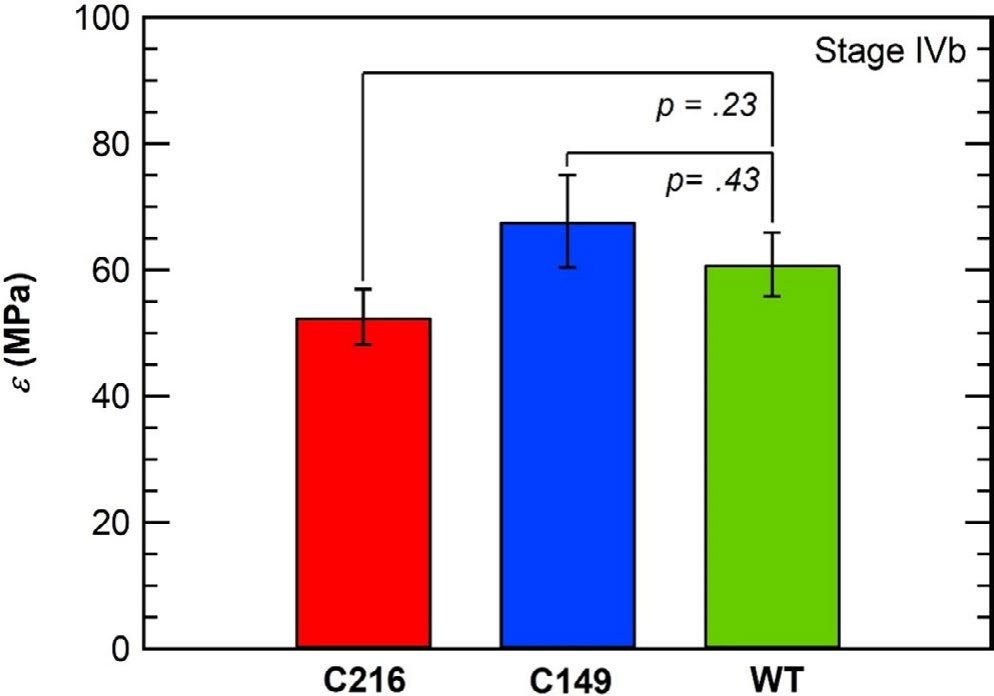
Comparison of mean *ε*‐values for (n) number of independent sporangiophore tested for wild type (WT) (*n* = 27) and stiff mutants (C216, *n* = 25 and C149, *n* = 18). The ε‐values represented are for stage IVb (growing) sporangiophores. *ε*‐values for stiff mutants were measured using in vivo turgor pressure step‐up experiments, while ε_‐_values for wild type were obtained from (Ortega, 2012). The vertical bars represent the standard error (*SEM*) of the mean value, and the horizontal bars enclose the area of statistical comparison for *p*‐values at 95% confidence interval

The mean *ε*‐values of stage IVb (growing sporangiophores) between wild type and stiff mutants are compared in Figure 6. The mean values for the C216 and C149 were measured at 52.6 ± *SEM* MPa and 67.7 ± *SEM* MPa, respectively; however, no significant difference was found for *ε*‐values between wild type and C149 (*p = .43*) using an unpaired *t* test. Furthermore, no significant difference was found for *ε*‐values between wild type and C216 (*p = .23*).

### 3.2 Dimensionless numbers Π_ev_, Π_pv _and Π_pe_

The biophysical variables *v*_s_ = (d*L*/d*t*)/*L* (steady relative volumetric growth rate), *ϕ* (relative longitudinal irreversible cell wall extensibility), *ε* (longitudinal volumetric elastic modulus), and *P*_c_ (critical turgor pressure) for wild type and stiff mutants C216 and C149 are shown in Table 1. All data for wild‐type sporangiophores were obtained from (Ortega, 2012) and (Ortega et al., 2012) published results. Stiff mutant data for variables *ϕ* and *P*_c_ are obtained from (Ortega et al., 2012) published results, and *ε* and *v*_s_ were measured in this study. Results from student *t* tests revealed that *ϕ* and *P*_c _were significantly different between wild type and both mutants (Ortega et al., 2012). The values in Table 1 are used to compute the magnitudes of the Π_ev_, Π_pv_, and Π_pe_. The maximum and minimum values for each variable in Table 1 were used to compute maximum and minimum values for the dimensionless parameters (see Appendix S2 for analysis).

**Table 1:** A comparison of relevant biophysical variables used in computing magnitudes of the Π_pe_, Π_pv_, and Π_ev_ dimensionless parameters: *ε*, *ϕ*, *P_c_*, and *ν_s_* for stage IVb sporangiophores for wild type, C216, and C149 strains

Variable units	Wild type Mean ± *SEM* (*n*)	C216 Mean ± *SEM* (*n*)	C149 Mean ± *SEM* (*n*)
*ε* (MPa)	60 ± 5.1 (27)	52.6 ± 4.4 (25)	67.7 ± 7.3 (18)
*ϕ* (min^−1^ MPa^−1^)	0.033 ± 0.005 (20)	0.012 ± 0.002 (18)	0.009 ± 0.002 (8)
*P*_c_ (MPa)	0.26 ± 0.01 (20)	0.13 ± 0.05 (18)	0.18 ± 0.08 (8)
*ν_s_* (min^−1^)	0.0011 ± 0.0001 (20)	0.0015 ± 0.0001 (59)	0.0015 ± 3.1 (59)

Note

The values are the mean ± the standard error (*SEM*) of (*n*) experiments. Values for *ν_s_*, ϕ, *ε*, and *P_c_* for wild type are obtained from (Ortega, 2012) and (Ortega et al., 2012). Values for ϕ and *P_c_* for stiff mutants are obtained from (Ortega et al., 2012), *ε* and *ν_s_* values were measured in this study.

**Figure 7:**
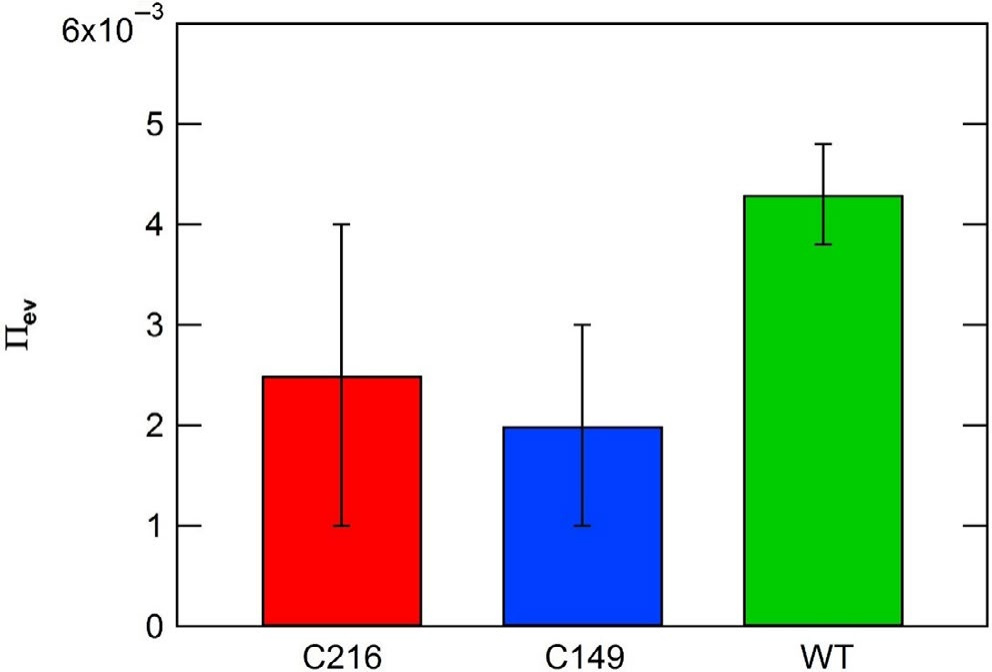
A comparison of the Π_ev_ values calculated for growing sporangiophores of *Phycomyces blakesleeanus* (stage IVb) for wild type (WT) and stiff mutants (C216 and C149). The maximum and minimum values (*SEM*) for *ε* and *P_c_*in Table 1 were used to compute maximum and minimum values, that is confidence intervals, for Π_ev_ using the same method as Ortega, 2017 (see Appendix S2 for calculations)

**Figure 8:**
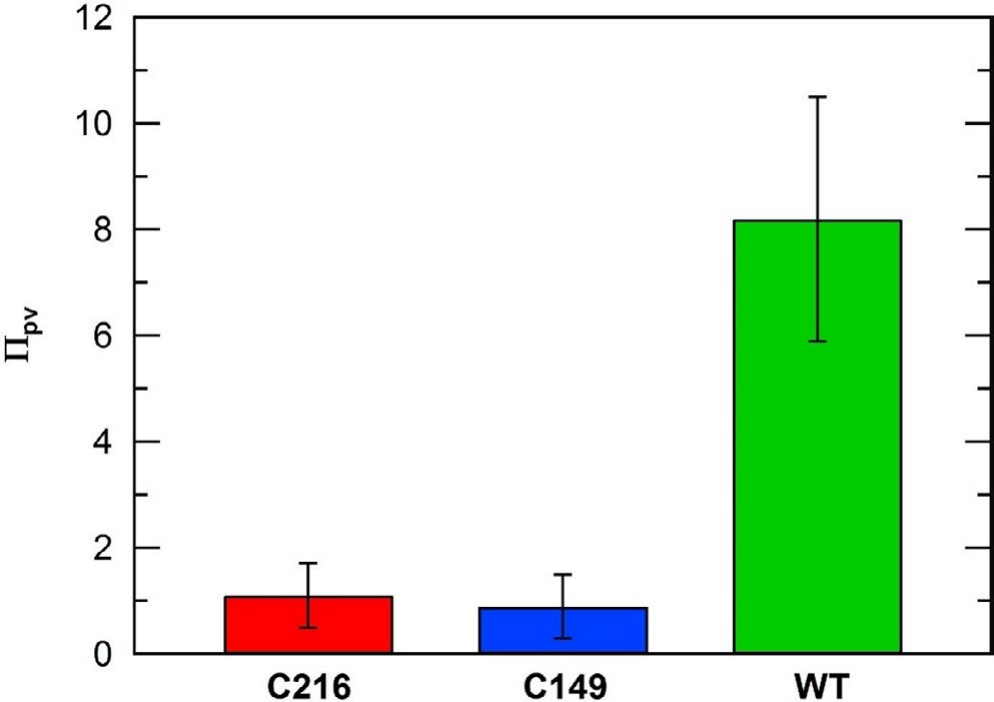
A comparison of the Π_pv_ values calculated for growing sporangiophores of *Phycomyces blakesleeanus *(stage IVb) for wild type (WT) and stiff mutants (C216 and C149). The maximum and minimum values (*SEM*) for *ϕ*, *P_c_*, and *ν_s_* in Table 1 were used to compute maximum and minimum values, that is confidence intervals, for Π_pv_ using the same method as Ortega, 2017 (see Appendix S2 for calculations)

**Figure 9:**
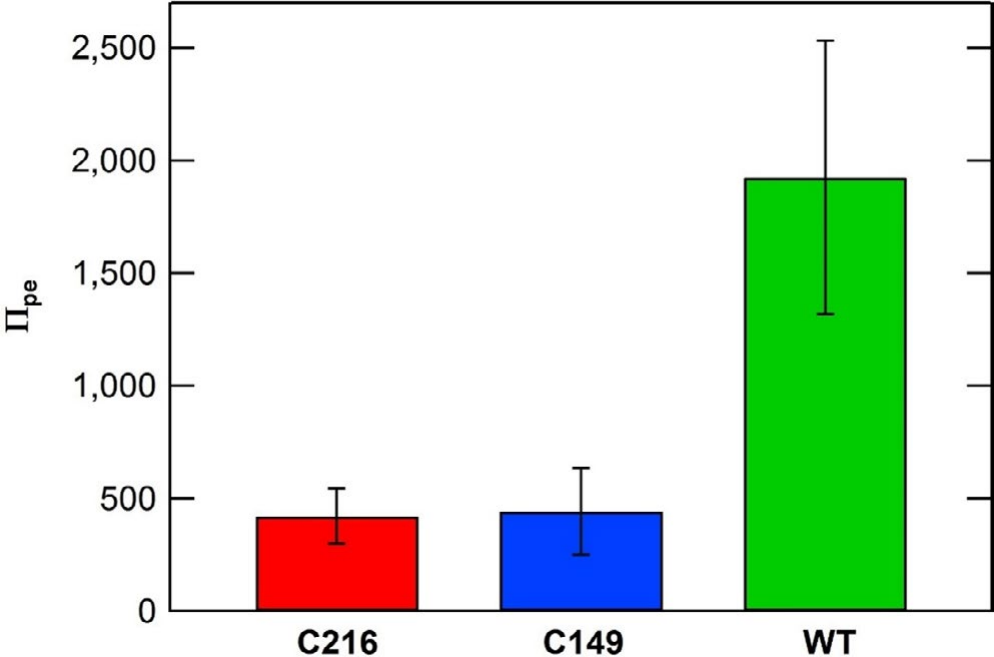
A comparison of the Π_pe_ values calculated for growing sporangiophores of *Phycomyces blakesleeanus* (stage IVb) for wild type (WT) and stiff mutants (C216 and C149). The maximum and minimum values (*SEM*) for *ϕ*, *ε*, and *ν_s_* in Table 1 were used to compute maximum and minimum values, that is confidence intervals, for Π_pe_ using the same method as Ortega, 2017 (see Appendix S2 for calculations)

The magnitudes of the Π_ev_, Π_pv_ *, *and Π_pe_ of wild type and stiff mutants were determined from in vivo creep and in vivo turgor pressure step‐up experiments conducted on intact stage IVb sporangiophores and are compared in Figures 7, 8, and 9. Details regarding the calculations of these parameters can be found in Appendix S2. The magnitudes of the Π_ev_ parameter were computed and compared to gain insight on the magnitude of elastic deformation rates in the wall for wild type and stiff mutants (Figure 7). The magnitudes of Π_ev_ for the stiff mutant sporangiophores are slightly smaller compared to those obtained from wild‐type sporangiophores. In contrast, the magnitudes of Π_pv _and Π_pe_ of stiff mutant sporangiophores are considerably smaller than respective values of wild‐type sporangiophores (Figures 8 and 9, respectively). The mean magnitude of Π_pv _for wild‐type sporangiophores is 7.5 times larger than the C216 mutant and 9.1 times larger than the C149 mutant (Figure 8). The mean magnitude of the Π_pe_ for wild‐type sporangiophores is 4.6 times larger than the C216 mutant and 4.4 times larger than the C149 mutant (Figure 9). The difference in the magnitudes Π_pv_ and Π_pe_ provides a comparison of the plastic deformation rates and stress relaxation rates between wild type and stiff mutants sporangiophores. Overall, plastic deformation rates and stress relaxation rates were substantially reduced in stiff mutants, while the elastic deformation rates of the wall were slightly reduced.

## 4 DISCUSSION

The Π_ev_ *,* Π_pv,_ and Π_pe_ dimensionless parameters were used to quantitate cell wall deformation rates and stress relaxation rates in two stiff mutants of the model organism *P. blakesleeanus* to gain insight on whether the mutant genes *madD*, *E*, *F*, *G*, and *J* genes affect these processes. The results provide insight into changes of wall deformation behavior and wall loosening chemistry of the stiff mutants. Prior experimental research showed that the magnitudes of the irreversible cell wall extensibility, *ϕ*, for stiff mutants C216 and C149 are significantly smaller than the wild type (Ortega et al., 2012), but no further evidence was obtained to learn whether the elastic and plastic wall deformation rates and stress wall relaxation rates were affected by the altered genes.

In this study, in vivo turgor pressure step‐up experiments were conducted to measure the longitudinal volumetric elastic modulus, *ε*, in stiff mutants to compute magnitudes for the Π_ev_ and Π_pe_ parameters. Previous data from in vivo creep experiments on stiff mutants and wild type that measured the relative irreversible cell wall extensibility, *ϕ*, and critical turgor pressure, *P*_c_, were used to compute the magnitudes of the Π_pv_ parameter for stiff mutants and wild type. Magnitudes of Π_ev_, Π_pv_, and Π_pe_ were compared between stiff mutants and wild type. The magnitudes of Π_pv_ and Π_pe_ were substantially smaller in stiff mutants compared to the wild type (Figures 8 and 9). It is noted that the magnitudes of Π_ev_, Π_pv_, and Π_pe_ are independent of the length of the cell or the length of the growth zone (see Appendix S3). The results demonstrate that the altered genes reduced plastic deformation rates and stress relaxation rates indicating that the cell wall loosening chemistry was significantly altered. A decrease in wall loosening chemistry can explain the observed diminished tropic responses exhibited by the stiff mutants. The magnitudes of the Π_ev_ were also computed for stiff mutants and wild type to learn if the cell wall elastic deformation rate was affected by the altered genes. The magnitudes of the Π_ev_ parameter were slightly smaller but similar to wild type (Figure 7). These results indicate that the wall elastic deformation rate did not substantially change and that the wall stiffness has not significantly changed with the altered genes. Noting this information, the mutants more accurately reflect “high viscosity” or “small wall extensibility” mutants (“wall viscosity” is the inverse of “wall extensibility”) instead of “stiff” mutants. The genetic mutation phenotype is not in “wall stiffness,” but in “wall viscosity” or “wall extensibility.”

If remodeling enzymes and/or proteins are responsible for cell wall loosening, it can be predicted from the results presented in this study that (a) there are fewer remodeling enzymes–proteins and/or (b) the activity of these cell wall remodeling enzymes–proteins is reduced in the stiff mutants. There is evidence that chitin synthases are involved in cell wall remodeling activity in *P. blakesleeanus* (Atsushi, Momany, Szaniszlo, Jayaram, & Tamotsu, 1993; Cubero, Ruiz‐Herrera, & Cerdá‐Olmedo, 1993; Jan, 1974). Jan (1974) demonstrated that chitin synthase activity in *P. blakesleeanus* sporangiophores was higher in the growing region (region where elongation takes place) compared to the non‐growing region. It was also shown that an increase in chitin synthase activity occurred after light stimulation that typically produces a transient increase in elongation growth rate, that is the light growth response. This increased chitin synthase activity could explain the light growth response if newly synthesized chitin produces wall loosening by competing for load‐bearing bonds. Previously, it was shown that stiff mutant sporangiophores have shorter growth zones compared to wild type (Ortega et al., 2012). It may be deduced that the stiff mutant sporangiophores have less chitin synthase activity due simply to having shorter growing zone, or the shorter growth zones are the result of less chitin synthase activity. Interestingly, Herrera‐Estrella and Ruiz‐Herrera (1983) labeled the reducing ends of chitin and showed an increase in reducing ends after light stimulation. Each poly‐GlcNAc chain in chitin contains only one reducing end, and therefore, an increase in reducing ends measures an increase in breaking of chitin microfibrils. Assuming the chitin microfibrils are load‐bearing polymers, the increase in chitin synthases activity could result in an increase in cell wall loosening and an increase in the plastic deformation rate of the cell wall. Most importantly, it was shown that *madB* and *madD* mutants did not show this increase in reducing ends. Previous studies such as these and the results presented in this study support the idea that chitin synthases may be involved in the rate of cell wall loosening, plastic deformation rate, and wall stress relaxation rate of the sporangiophore of *P. blakesleeanus*. If chitin synthases are involved in wall loosening, the results presented here predict that the stiff mutants have a smaller amount chitin synthases and/or a lower chitin synthase activity.

The prediction that arises from the results of this study can further be investigated to learn if the chitin synthase activity in stiff mutants is lowered. The amount of chitin synthase activity can be indirectly accounted for by estimating the number of chitosomes in stiff mutants and wild type, because it has been shown that chitosomes carry 80% of chitin synthase activity in a fungal cell wall (Ruiz‐Herrera, Bracker, & Bartnicki‐Garcia, 1984). To learn if chitin synthase activity is lowered in stiff mutants, the in vitro activation of chitosomes in stiff mutants can be investigated by similar methods used by Herrera‐Estrella, Chavez, and Ruiz‐Herrera (1982) and Martinez‐Cadena and Ruiz‐Herrera (1987).

Another important area of investigation is with chitin synthase mutants. Currently, there is no evidence to report whether these mutants are affected in cell wall elastic or plastic deformation rates and stress relaxation rates. Determining the magnitude of the relevant dimensionless numbers of these mutants can help gain more insight into cell wall remodeling, morphology, and expansive growth in *Phycomyces blakesleeanus* and other fungi.

The dimensionless numbers presented in this study can be used to quantify wall elastic and plastic deformation rates, and stress relaxation rates to make a direct comparison between wild type and chitin synthase mutants or any other mutant and gain insights into the genes linked to these mutations.

It is possible to use the dimensionless numbers as a way to rule out genes, proteins, and enzymes that do not produce the expected wall loosening chemistry, wall stress relaxation rates, and wall deformation rates observed in wall expansive growth. The dimensionless numbers can help gain better insight into how the cell wall is affected under different conditions such as environmental stresses and gene modification. Furthermore, these methods can be conducted on in vivo cells, thus ensuring that the cell wall is operating in normal living and growing conditions. Finally, the methods presented in this study can help optimize cell walls for the use in industrial and pharmaceutical sectors.

## 5 CONCLUSIONS

The results presented in this study show that the *madD*, *E*, *F*, *G*, and *J* genes only substantially affect irreversible (plastic) deformation rates and stress relaxation rates, central processes in expansive growth in stiff mutants of *P. blakesleeanus*. These results indicate that the cell wall loosening chemistry is altered so that less cell wall loosening occurs in the stiff mutants. The results suggest that the diminished tropic responses exhibited by the stiff mutants are due to an alteration in the cell wall loosening chemistry by the *madD*, *E*, *F*, *G*, and *J* genes. It is concluded that the mutant sporangiophores are not stiffer and that the term “stiff” mutant is not an appropriate indicator of the change of cell wall properties.

## 6 FUTURE RESEARCH: DIMENSIONLESS NUMBERS FOR WATER UPTAKE AND TRANSPIRATION

In the study conducted by Ortega et al. (2012), it was found that stiff mutants and wild‐type sporangiophores exhibit similar growth rates. However, the relative cell wall extensibility, *ϕ*, decreased in mutants while the effective wall stresses, (*P*−*P*_c_), increased. It was concluded that in stiff mutants, the higher wall stresses produced by higher turgor pressure compensate for the lower wall extensibility to maintain similar elongation growth rates as wild‐type sporangiophores. This result suggests that *L*_p_ may increase. Also, it is not known if the transpiration rates of these mutant sporangiophores are larger, smaller, or about the same as those measured in wild‐type sporangiophores. Future research will be conducted to measure the magnitude of *L*_p_ and transpiration rate of these mutant sporangiophores to learn if they change in magnitude. In doing so, the magnitude of Π_wv_ and Π_Tv_ can be determined to learn if they change in magnitude. The determination of the magnitudes of Π_wv_ and Π_Tv_ will allow the comparison of the magnitudes of “*net water uptake rate*” (Π_wv_ ‐ Π_Tv_), and “*total wall deformation rate*” (Π_pv_ + Π_ev_), by determination of the dimensionless number, Π_wd_ (Ortega, 2019).

$$\prod_{\text{wd}}=\left(\frac{\prod_{\text{wv}}-\prod_{\text{Tv}}}{\prod_{\text{pe}}+\prod_{\text{ev}}}\right)$$

It was found that for wild‐type stage IV sporangiophores, the ratio of net water uptake rate to total wall deformation rate, that is Π_wd_, is approximately 16.3. This magnitude indicates that the sporangiophore's capability of net water uptake rate is 16 times larger than the capability of its cell wall to deform. Thus, the limiting biophysical process for expansive growth of the wild‐type stage IV sporangiophore is the wall deformation rate. In the future, experiments measuring hydraulic conductivity, *L*_p_, of the plasma membrane and transpiration rate will be conducted to determine the magnitudes of Π_wv_, Π_Tv_, and Π_wd_ for the stiff mutants. The accompanying quantitative analysis can help determine which biophysical processes are significantly altered by the mutant genes and by how much. Furthermore, this type of quantitative analysis can help provide insight into which biophysical process is the limiting process in expansive growth of stiff mutants.

## CONFLICT OF INTEREST

The authors declare no conflict of interest associated with the work described in this article.

## AUTHOR CONTRIBUTIONS

CMM developed the experimental protocol, conducted the experiments, and data analysis. JKEO helped design the research and provided guidance on experimental protocols, data analysis and data interpretation. Both authors contributed equally to writing the manuscript
