# Supplementary material for: Dimensionless numbers to study cell wall deformation of stiff mutants of Phycomyces blakesleeanus
Source: Plant Direct. 2019 Dec 27;3(12):e00195. doi: 10.1002/pld3.195 (PMC6933610; doi:10.1002/pld3.195)
Supplement: Supplementary file 1 [file PLD3-3-e00195-s001.docx]

**Appendix 1**

Nomenclature used in this study:

$A=$ *area of the plasma membrane*

$L=$*length of the cell (sporangiophore)*

$L_{P}=$*hydraulic conductivity of the plasma membrane*

$V=$ *volume*

$L_{w}=\left( \frac{L_{p}A}{V} \right)=$*relative hydraulic conductance of the plasma membrane*

$P=$*turgor pressure (gage pressure) relative to the atmosphere*

$P_{C}=$*critical turgor pressure (to be exceeded before plastic extension begins)*

$t=$ *time*

$V_{\mathrm{cw}}=$*volume of the cell wall chamber*

$V_{w}=$*volume of water in the cell*

$V_{T}=$*volume of water lost through transpiration*

$v=\left( \frac{{dV}}{V {dt}} \right)=$*relative rate of change in volume of the cell*

$v_{\mathrm{cw}}=\left( \frac{{dV}_{\mathrm{cw}}}{V_{\mathrm{cw}} {dt}} \right)=$*relative rate of change in volume of the cell wall chamber*

${v_{s}}=\left( \frac{{dV}}{V {dt}} \right)=$*steady or quasi-steady relative rate of change in volume of the cell*

$v_{T}=\left( \frac{{dV}_{T}}{V_{T} {dt}} \right)=$*relative rate of change in water volume lost via transpiration*

$v_{w}=\left( \frac{{dV}_{w}}{V_{w} {dt}} \right)=$*relative rate of change in water volume in the cell*

$\varepsilon=$*volumetric elastic modulus of the cell wall*

$\phi=$*relative irreversible extensibility of the cell wall*

*m= longitudinal irreversible wall extensibility*

$\Pi=$ *dimensionless number*

$\Delta\pi=$*osmotic pressure difference across the plasma membrane*

*L_w_* (*Δπ – P*) *= relative volumetric rate of water uptake*

*φ* (*P – P*_C_) *= relative volumetric plastic deformation rate of the cell wall*

$\frac{{dP}}{\varepsilon{} {dt}}$*= relative volumetric elastic deformation rate of the cell wall*

**Appendix 2**

Computation of Π Dimensionless Parameters

The same process was used as in (Ortega, 2016) to compute stiff mutant Π-values. The process consists of computing the largest Π-value and the smallest Π-value to obtain an average Π-value. For reference, Ortega’s computation of the Π_pe_ value for intact wild type sporangiophores is presented. All other Π-values for wild type and stiff mutants are computed here as part of the study presented.

*Wild Type* Π-*Values*

The following values were obtained for intact stage IVb wild type sporangiophores of *Phycomyces blakesleeanus*. Values for variables *L, dL/dt*, *m,* *P*_c_ and *ε* were obtained from (Ortega, 2012, 2016, 2017):

Wild Type Π_pe_:

$$L=3 X {10}^{4} \mu m$$

$$\frac{dL}{dt}=34\pm3.1 \left( SE \right) \mu m {min}^{-1} (n=20)$$

$$v_{s}=\frac{1}{L}\frac{dL}{dt}=0.0011\pm0.0001 \left( SE \right) {min}^{-1} (n=20)$$

$$v_{s} \left( largest \right)=\left( 0.0011+0.005 \right)=0.0012 {min}^{-1}=0.072 h^{-1}$$

$$v_{s} \left( smallest \right)=\left( 0.0011-0.005 \right)=0.0010 {min}^{-1}=0.060 h^{-1}$$

$m=997\pm160 \left( SE \right)$ $\mu m {min}^{-1}{MPa}^{-1} (n=20)$

$$\phi=\frac{m}{L}=0.033 \pm0.005 \left( SE \right) {min}^{-1}{MPa}^{-1} (n=20)$$

$$\phi\left( largest \right)=\left( 0.033+0.005 \right)=0.038 {min}^{-1}{MPa}^{-1}=2.3 h^{-1}{MPa}^{-1}$$

$$\phi\left( smallest \right)=\left( 0.033-0.005 \right)=0.028 {min}^{-1}{MPa}^{-1}=1.7 h^{-1}{MPa}^{-1}$$

$$\varepsilon=60.9\pm5.1 \left( SE \right) MPa (n=27)$$

$${}_{\mathrm{pe}}\left( largest \right)=\left( \frac{\varepsilon\phi(largest)}{v_{s}(smallest)} \right)=\left( \frac{(66 MPa)(2.3 h^{-1}{MPa}^{-1})}{{0.060 h}^{-1}} \right)\approx2530$$

$${}_{\mathrm{pe}}\left( smallest \right)=\left( \frac{\varepsilon\phi\left( smallest \right)}{v_{s}\left( largest \right)} \right)=\left( \frac{\left( 55.8 MPa \right)\left( 1.7 h^{-1}{MPa}^{-1} \right)}{{0.072 h}^{-1}} \right)\approx1318$$

$${}_{\mathbf{pe}}\boldsymbol{(WT)\approx}\boldsymbol{1924 \pm606}$$

- - - 1. Wild Type _pv_:

$${}_{\mathrm{pv}}\left( largest \right)=\left( \frac{\phi P_{c}\left( largest \right)}{v_{s}\left( smallest \right)} \right)=\left( \frac{(2.3 h^{-1}{MPa}^{-1})(0.26+0.01 MPa)}{{0.062 h}^{-1}} \right)\approx10.4$$

$${}_{\mathrm{pv}}\left( smallest \right)=\left( \frac{\phi P_{c}\left( smallest \right)}{v_{s}\left( largest \right)} \right)=\left( \frac{(1.7 h^{-1}{MPa}^{-1})(0.26-0.01 MPa)}{{0.072 h}^{-1}} \right)\approx5.9$$

$${}_{\mathbf{pv}}\left( \mathbf{WT} \right)\boldsymbol{\approx}\boldsymbol{8.2 \pm2.3}$$

- - - 1. Wild Type _ev_:

$${}_{\mathrm{ev}}\left( largest \right)=\frac{P_{c}(largest)}{\varepsilon(smallest)}=\frac{(0.26+0.01 MPa)}{(60.9-5.1 MPa)}\approx0.0048$$

$${}_{\mathrm{ev}}\left( smallest \right)=\frac{P_{c}(smallest)}{\varepsilon(largest)}=\frac{(0.26-0.01 MPa)}{(60.9+5.1 MPa)}\approx0.0038$$

$${}_{\mathbf{ev}}\boldsymbol{(WT)\approx}\boldsymbol{0.0043 \pm0.0005}$$

*C216* -*values*

The following values were obtained for intact stage IVb C216 sporangiophores of *Phycomyces blakesleeanus*. Values for variables *L, dL/dt,* and *ε*  were determined in the study presented and values for *m* and *P*_c_ were obtained from (Ortega et al., 2012):

- - - 1. C216 _pe_*:*

$$L=1.8 X {10}^{4} \mu m$$

$$\frac{dL}{dt}=27.3\pm2.5 \left( SE \right) \mu m {min}^{-1} (n=59)$$

$$v_{s}=\frac{1}{L}\frac{dL}{dt}=0.0015\pm0.0001 \left( SE \right) {min}^{-1} (n=59)$$

$$v_{s}\left( largest \right)=\left( 0.0015+0.0001 \right)=0.0016 {min}^{-1}=0.096 h^{-1}$$

$$v_{s}\left( smallest \right)=\left( 0.0015-0.0001 \right)=0.0014 {min}^{-1}=0.084 h^{-1}$$

$m=222\pm40 \left( SE \right)$ $\mu m {min}^{-1}{MPa}^{-1} (n=18)$

$$\phi=\frac{m}{L}=0.012\pm0.002 \left( SE \right) {min}^{-1}{MPa}^{-1} (n=18)$$

$$\phi\left( largest \right)=\left( 0.012+0.002 \right)=0.014 {min}^{-1}{MPa}^{-1}=0.84 h^{-1}{MPa}^{-1}$$

$$\phi\left( smallest \right)=\left( 0.012-0.002 \right)=0.010 {min}^{-1}{MPa}^{-1}=0.60 h^{-1}{MPa}^{-1}$$

$$\varepsilon=52.6 \pm4.4 \left( SE \right) MPa (n=25)$$

$${}_{\mathrm{pe}}\left( largest \right)=\left( \frac{\varepsilon\phi(largest)}{v_{s}(smallest)} \right)=\left( \frac{(57 MPa)(0.84 h^{-1}{MPa}^{-1})}{{0.084 h}^{-1}} \right)\approx543$$

$${}_{\mathrm{pe}}\left( smallest \right)=\left( \frac{\varepsilon\phi(smallest)}{v_{s}(largest)} \right)=\left( \frac{(48.2 MPa)(0.60 h^{-1}{MPa}^{-1})}{{0.096 h}^{-1}} \right)\approx301$$

$${}_{\mathbf{pe}}\boldsymbol{(C216)\approx}\boldsymbol{422 \pm121}$$

- - - 1. C216 _pv_*:*

$${}_{\mathrm{pv}}\left( largest \right)=\left( \frac{\phi P_{c}\left( largest \right)}{v_{s}\left( smallest \right)} \right)=\left( \frac{(0.84 h^{-1}{MPa}^{-1})(0.13+0.05 MPa)}{{0.084 h}^{-1}} \right)\approx1.7$$

$${}_{\mathrm{pv}}\left( smallest \right)=\left( \frac{\phi P_{c}\left( smallest \right)}{v_{s}\left( largest \right)} \right)=\left( \frac{(0.60 h^{-1}{MPa}^{-1})(0.13-0.05 MPa)}{{0.096 h}^{-1}} \right)\approx0.5$$

$${}_{\mathbf{pv}}\left( \mathbf{C216} \right)\boldsymbol{\approx1 .1\pm0.6}$$

- - - 1. C126 _ev_*:*

$${}_{\mathrm{ev}}\left( largest \right)=\frac{P_{c}(largest)}{\varepsilon(smallest)}=\frac{(0.13+0.05 MPa)}{(52.6-4.4 MPa)}\approx0.0010$$

$${}_{\mathrm{ev}}\left( smallest \right)=\frac{P_{c}(smallest)}{\varepsilon(largest)}=\frac{(0.13-0.05 MPa)}{(52.6+4.4 MPa)}\approx0.0037$$

$${}_{\mathbf{ev}}\left( \mathbf{C216} \right)\boldsymbol{\approx0.0025 \pm0.0015}$$

*C149 -values*

The following values were obtained for intact stage IVb C149 sporangiophores of Phycomyces blakesleeanus. Values for variables *L*, d*L*/d*t*, and *ε* were determined in the study presented and values for *m* and *P*_c_ were obtained from (Ortega et al., 2012):

- - - 1. C149 _pe_:

$$L=1.8 X {10}^{4} \mu m$$

$$\frac{dL}{dt}=27.8\pm3.1 \left( SE \right) \mu m {min}^{-1} (n=59)$$

$$v_{s}=\frac{1}{L}\frac{dL}{dt}=0.0015\pm0.0002 \left( SE \right) {min}^{-1}(n=59)$$

$$v_{s}\left( largest \right)=\left( 0.0015+0.0002 \right)=0.0017 {min}^{-1}=0.102 h^{-1}$$

$$v_{s}\left( smallest \right)=\left( 0.0015-0.0002 \right)=0.0013 {min}^{-1}=0.078 h^{-1}$$

$m=170\pm30 \left( SE \right)$ $\mu m {min}^{-1}{MPa}^{-1} (n=8)$

$$\phi=\frac{m}{L}=0.009\pm0.002 \left( SE \right) {min}^{-1}{MPa}^{-1} (n=8)$$

$$\phi\left( largest \right)=\left( 0.009+0.002 \right)=0.011 {min}^{-1}{MPa}^{-1}=0.66 h^{-1}{MPa}^{-1}$$

$$\phi\left( smallest \right)=\left( 0.009-0.002 \right)=0.007 {min}^{-1}{MPa}^{-1}=0.42 h^{-1}{MPa}^{-1}$$

$$\varepsilon=67.7\pm7.3 \left( SE \right) MPa (n=18)$$

$${}_{\mathrm{pe}}\left( largest \right)=\left( \frac{\varepsilon\phi(largest)}{v_{s}(smallest)} \right)=\left( \frac{(75 MPa)(0.66 h^{-1}{MPa}^{-1})}{{0.078 h}^{-1}} \right)\approx635$$

$${}_{\mathrm{pe}}\left( smallest \right)=\left( \frac{\varepsilon\phi(smallest)}{v_{s}(largest)} \right)=\left( \frac{(60.4 MPa)(0.42 h^{-1}{MPa}^{-1})}{{0.102 h}^{-1}} \right)\approx249$$

$${}_{\mathbf{pe}}\boldsymbol{(C149)\approx}\boldsymbol{442 \pm193}$$

- - - 1. C149 _pv_:

$${}_{\mathrm{pv}}\left( largest \right)=\left( \frac{\phi P_{c}\left( largest \right)}{v_{s}\left( smallest \right)} \right)=\left( \frac{(0.66 h^{-1}{MPa}^{-1})(0.18+0.08 MPa)}{{0.078 h}^{-1}} \right)\approx1.5$$

$${}_{\mathrm{pv}}\left( smallest \right)=\left( \frac{\phi P_{c}\left( smallest \right)}{v_{s}\left( largest \right)} \right)=\left( \frac{(0.42 h^{-1}{MPa}^{-1})(0.18-0.08 MPa)}{{0.102 h}^{-1}} \right)\approx0.3$$

$${}_{\mathbf{pv}}\left( \mathbf{C149} \right)\boldsymbol{\approx0.9 \pm0.6}$$

- - - 1. C149 _ev_:

$${}_{\mathrm{ev}}\left( largest \right)=\frac{P_{c}(largest)}{\varepsilon(smallest)}=\frac{(0.18+0.08 MPa)}{(67.7-7.3 MPa)}\approx0.0010$$

$${}_{\mathrm{ev}}\left( smallest \right)=\frac{P_{c}(smallest)}{\varepsilon(largest)}=\frac{(0.18-0.08 MPa)}{(67.7+7.3 MPa)}\approx0.0029$$

$${}_{\mathbf{ev}}\left( \mathbf{C149} \right)\boldsymbol{\approx}\boldsymbol{0.0020 \pm0.0010}$$

**Appendix 3 – Analysis showing that the magnitudes of Π_ev,_ Π_pv,_ and Π_pe_ are independent of characteristic length, *L*_c_, where *L*_c_ = *L* or *L*_c_ = *L*_gz_.**

Consider the magnitudes of the dimensionless parameters Π_ev,_ Π_pv,_ and Π_pe_:

$${}_{\mathrm{ev}}=\frac{P_{c}}{\varepsilon}$$

$${}_{\mathrm{pv}}=\frac{\phi P_{c}}{v_{s}}$$

$${}_{\mathrm{pe}}=\frac{\varepsilon\phi}{v_{s}}$$

where the relative irreversible wall extensibility, 𝜙, is equal to the wall extensibility, *m*, divided by the characteristic length, *L*_c_, therefore 𝜙= *m*/*L*_c_. The relative elongation rate, *v*_s_, is equal to the elongation rate, d*L*/d*t*, divided by the characteristic length, *L*_c_, therefore *v*_s_=(d*L*/d*t*)/*L*_c_. Substituting these into the dimensionless parameters Π_pv_ and Π_pe,_ these become,

$${}_{\mathrm{pv}}=\frac{(\frac{m}{L_{c}})P_{c}}{(\frac{dL}{dt})/L_{c}}=\frac{\boldsymbol{m}\boldsymbol{P}_{\boldsymbol{c}}}{\boldsymbol{dL/dt}}$$

$${}_{\mathrm{pe}}=\frac{\varepsilon(\frac{m}{L_{c}})}{(\frac{dL}{dt})/L_{c}}=\frac{\boldsymbol{\varepsilon m}}{\boldsymbol{dL/dt}}$$

and Π_ev_ is independent of $\phi$ and *v*_s_.
