# Supplementary material for: Dimensionless numbers to study cell wall deformation of stiff mutants of Phycomyces blakesleeanus
Source: Plant Direct. 2019 Dec 27;3(12):e00195. doi: 10.1002/pld3.195 (PMC6933610; doi:10.1002/pld3.195)
Supplement: Supplementary file 2 [file PLD3-3-e00195-s002.pdf]

November 24, 2019

Re: Resubmission of manuscript *Dimensionless Numbers to Study Cell Wall Deformation of Stiff Mutants of Phycomyces blakesleeanus*, 2019-00294-T2

Dr. Ivan Baxter
Editor in Chief
Plant Direct
4-NW-131 Station Landing
111 River Street, Hoboken NJ 07030

Dear Dr. Baxter,

Thank you again for the opportunity to revise our manuscript, *Dimensionless Numbers to Study Cell Wall Deformation of Stiff Mutants of Phycomyces blakesleeanus*. We would like to thank the reviewer for the through, comprehensive and thoughtful review. We believe the additions and edits in this second review have substantially improved the manuscript.

The manuscript has been revised to address the reviewer's comments, which are appended alongside our responses to this letter. Our responses are in blue type and italics, including how and where the text was modified. Changes made in the manuscript are marked using track changes. You will find the clean version of the latest version of the manuscript uploaded alongside this document. The revision has been developed in consultation with the coauthor, and each author has given approval to the final form of this revision.

We very much hope the revised manuscript is accepted for publication in *Plant Direct*.

Sincerely yours,

Dr. Cindy Munoz
Dr. Joseph K.E. Ortega

Reviewer #1:

The authors have largely address my concerns, particularly about the formulation of equations, scope of data interpretation and clarity of statistics. I remain interested in the manuscript's central message that changes in cell wall extensibility but not elasticity is sufficient to explain the growth rate phenotypes of the promptly redefined "viscosity" or "extensibility" mutants of P. blakesleeanus, and that wall extensibility and elasticity are uncoupled in the studied fungal cell wall. This conclusion partially concides with the recent works from the Cosgrove lab, where different mechanical properties were also found to be not necessarily coupled in plant cell walls (Zhang et al., 2019 Plant J.; Wang and Cosgrove, 2019 preprint). This manuscript, as well as other complementary studies, will update the current understanding of the biomechanics of walled-cell expansion.
I however would still like the authors to address the remaining clarity issues (all the line numbers are based on the tracked change version).

1. Confusion of L: L is defined as "length of the cell (sporangiophore)" in Appendix 1, while Fig. 2 indicated that delta_L is "change in elongation" (so L is elongation). This can be confusing, particularly since Fig. 2B starts with L = 0 um (presumably cell length is 3 * 10^4 um, not 0 um. Delta_L should start at 0 um, right?). The authors must clearly define and correctly annotate L and delta_L, in the whole manucript and particularly in the Fig. 2 legend and Fig. 2 axes.

We agree with the reviewer in that this annotation is confusing. We have edited the figure and text to better represent the measured length and the cell's initial length. We have added the L_{ref} annotation to represent length changes measured when turgor pressure step-ups began. L_{ref} excludes the initial cell's length. Figure 2B should begin at L_{ref} =0 um, this graphical error is now fixed and can be seen in the updated Figure 2B. Lines 232-235, 237-238. We have edited the manuscript to reflect this change as well, lines 462-463, 512-514.

2. L and the constant growth zone length: as I questioned in the previous round, the constant growth zone length (~ 3.5 um at stage VIb, inferred from Fig. 1) is much smaller than the cell length (30 mm). This means that the absolute magnitude of the studied dimensionless number will be very different depending on whether the cell length or growth zone length are considered. Indeed the effect of L is partially cancelled by phi/v_s in PI_pe and PI_pv, and finally the fold change between genotypes (e.g. PI_pe/PI_pe between WT and C216) completely cancels out L in epsilon, too (if both cell length and growth zone length are indifferent between genotypes). The authors should consider to reformulate the numerical computation of PI parameters by cancelling out L first before bringing in

numbers, and use the ratio of PI instead of PI themselves as the reporting values. Alternatively the authors should discuss about the choice of L in regard to the "constant growth zone length".

The reviewer states; "that the absolute magnitude of the studied dimensionless number will be very different depending on whether the cell length or growth zone length are considered". This may appear to be the case, but the magnitudes of the dimensionless numbers are independent of the characteristic length, L_c, used. Consider the magnitude of the dimensionless number $\Pi_{pe} = (\varepsilon\phi/v_s)$. First, the relative irreversible wall extensibility, ϕ, is equal to the wall extensibility, m, divided by the characteristic length, L_c, so $\phi = m/L_c$. Second, the relative elongation rate, v_s, is equal to the elongation rate, dL/dt, divided by the characteristic length, L_c, so $v_s = (dL/dt)/L_c$. Substituting into the dimensionless number, $\Pi_{pe} = \varepsilon\phi/v_s$, we get $\Pi_{pe} = \varepsilon(m/L_c)/ (dL/dt)/L_c = \varepsilon m/(dL/dt)$. The same analysis can be used to show that the magnitude of Π_{pv} is independent of the characteristic length, L_c, i.e. $\Pi_{pv} = \phi P_C/v_s = [(m/L_c)P_C]/ [(dL/dt)/L_c] = mP_C/(dL/dt)$. Thus the mathematical expressions, $\Pi_{pe} = \varepsilon m/(dL/dt)$ and $\Pi_{pv} = mP_C/(dL/dt)$, demonstrate that the magnitudes of Π_{pe} and Π_{pv} are independent of L_c and only depend on the magnitude of ε, m, P_C and the elongation rate, dL/dt. In other words, the magnitudes of Π_{pe} and Π_{pv} are independent of whether the "length of the cell" or the "length of the growth zone" is used as the characteristic length. However, the dimensionless numbers were derived from global biophysical equations that were derived for the whole cell. Thus the magnitude of the biophysical variables used in the relevant dimensionless numbers must be for the whole cell, i.e. the length of the whole cell, L, is used as the characteristic length. We understand that this can be a point of confusion so we added a sentence in the Discussion to refer the reader to Appendix 3 (pgs 668-681) that explicitly addresses this concern.

Other minor comments:
Line 99: Stems, roots and leaves are not higher plants, but higher plant organs.

Fixed, line 90

Line 152-162: Indeed neither water uptake nor turgor pressure can cause changes in bending, they may still contribute to "the magnitude[s] of plastic deformation rate" as in line 160, and is also expressed in Equation 2 and in the dimensionless number PI_we. The authors should consider to shortly discuss about the contributions of water-related dimensionless parameters (their necessity or otherwise) in interpreting the reduced growth rate phenotype.

Thank you for making this observation. We have added a new section "Future Research: Dimensionless numbers for water uptake and transpiration" to address this (see lines 423-450).

Line 183-185: Consider to shorten the sentence by removing potential repeats.

Removed repeated text, lines 152-154. Added stiffness to emphasize why the volumetric modulus, ε, was found, line 155.

Line 605: L should be L_w.

Corrected, line 580

Appendix 2 line 620, 627 & 635: correct unit for dL/dt.

Corrected, lines 596, 622, and 648.

Fig. 9: Consider to define the red line and red arrow in figure legend to help readers not familiar with pressure probe experiments.

Edited, lines 552-554. Edited Figure 7, line 496.
